# Automated *In Vivo* Platform for the Discovery of Functional Food Treatments of Hypercholesterolemia

**DOI:** 10.1371/journal.pone.0052409

**Published:** 2013-01-21

**Authors:** Robert M. Littleton, Kevin J. Haworth, Hong Tang, Kenneth D. R. Setchell, Sandra Nelson, Jay R. Hove

**Affiliations:** 1 Department of Molecular and Cellular Physiology, University of Cincinnati College of Medicine, Cincinnati, Ohio, United States of America; 2 Molecular and Developmental Biology, Cincinnati Children's Hospital Medical Center, Cincinnati, Ohio, United States of America; 3 Division of Cardiovascular Diseases, Department of Internal Medicine, University of Cincinnati College of Medicine, Cincinnati, Ohio, United States of America; 4 Drug Discovery Center, The University of Cincinnati, Cincinnati, Ohio, United States of America; 5 Division of Pathology and Laboratory Medicine, Cincinnati Children's Hospital Medical Center, Cincinnati, Ohio, United States of America; The Centre for Research and Technology, Hellas, Greece

## Abstract

The zebrafish is becoming an increasingly popular model system for both automated drug discovery and investigating hypercholesterolemia. Here we combine these aspects and for the first time develop an automated high-content confocal assay for treatments of hypercholesterolemia. We also create two algorithms for automated analysis of cardiodynamic data acquired by high-speed confocal microscopy. The first algorithm computes cardiac parameters solely from the frequency-domain representation of cardiodynamic data while the second uses both frequency- and time-domain data. The combined approach resulted in smaller differences relative to manual measurements. The methods are implemented to test the ability of a methanolic extract of the hawthorn plant (*Crataegus laevigata*) to treat hypercholesterolemia and its peripheral cardiovascular effects. Results demonstrate the utility of these methods and suggest the extract has both antihypercholesterolemic and postitively inotropic properties.

## Introduction

Americans are increasingly turning to natural product treatments of hypercholesterolemia, yet research to develop natural products as pharmaceuticals has declined in recent years [1], [2], [3]. This is due in part to the emphasis in modern drug discovery on single molecule treatments; when plant material is fractionated to the molecular level, the beneficial effects of the plant are often lost [4]–[6], [2]. However, plant material extracts present an exceedingly large number of natural products to test. Therefore an automated system capable of high-throughput screening would be of benefit.

While there are many cell-culture systems amenable to high-throughput, automated screening, cell-based assays yield effectors of specific molecules and pathways that do not necessarily translate to clinical efficacy due to dissimilar physiology compared to humans. Mammalian models, while physiologically similar to humans, present difficulty when assaying large numbers of organisms[7]. The zebrafish model strikes an ideal balance between cell-culture and mammalian assay systems, having complex vertebrate organ systems including cardiovascular, nervous and enteric systems. The relative simplicity and small size of these organ systems, along with high fecundity make analyzing them in a high throughput manner readily accessible [8]. Zebrafish models also present the opportunity to assay for food-based disease treatments with organ system genetics as well as physiology that displays remarkable similarity to the human condition [9].

The transparency and small size of the embryonic zebrafish allows microscopic visualization and quantification of fluorescent lipids within vertebrate organ systems. Several studies have taken advantage of this prospect to investigate fundamental mechanisms of lipid metabolism as well as test for new treatments that alter lipid absorption [10], [11]. With respect to hypercholesterolemia, larval zebrafish fed a high-cholesterol diet (HCD) have increased endothelial layer thickening and disorganization, vascular leukocyte recruitment, vascular leakage, and vascular neutral fat deposition [12]. zetimibe treatment resolved endothelial thickening, disorganization and leakage due to an HCD. HCD-fed larval zebrafish also have a 4-fold increase in total cholesterol and triglycerides, a 10–70× increase in cholesterylesters, and increased levels of ApoB and ApoAI [13]. Therefore, lipid profiles, lipid level alterations, immunological response and vascular changes associated with an HCD in zebrafish are similar to those seen in mammalian models of atherosclerosis. Besides numerous studies demonstrating that treatment of zebrafish with antihyperlipidemic drugs mirrors the response of humans to those drugs [14], [15], scientists are also beginning to test the ability of natural products to treat hypercholesterolemia. In the adult zebrafish, turmeric, laurel, cinnamon and clove reduced blood serum lipid and cholesterol levels [16], [17]. Additionally, BODIPY- cholesterol (BOD-CH) has been established as a marker of intravascular cholesterol levels in the zebrafish and it was demonstrated that ground hawthorn leaves and flowers administered in the diet decrease intravascular BOD-CH fluorescence in zebrafish larvae [18].

Until recently, the ability to test natural product treatments in a food-based treatment paradigm via high-throughput screening has not been possible [2]. Here we develop and test an automated, zebrafish-based hypercholesterolemia treatment screen focused on natural product drug discovery and amenable to high-throughput testing, which can also be utilized to test the efficacy of purified molecular pharmaceuticals. We utilize this method to test the ability of a methanolic hawthorn (*Crataegus laevigata*) leaf and flower extract (MHE) to impact hypercholesterolemia.

Analyzing time varying cardiac variables is one of the most valuable assessments of a treatment' overall physiological effects [19]. A treatment that influences cardiac function impacts flow throughout the entire organism. Manually analyzing and quantifying these data sets is time consuming. Further, making measurements on large numbers of organisms creates a significant amount of data to be analyzed. Depending on the complexity of data analysis, manual techniques can be tedious, do not take into account the entirety of the acquired time varying data, o may be prone to subjective biases. We have developed an automated system for analyzing high-speed confocal data of the zebrafish heartbeat, resulting in rapid analysis. We utilize our method to test the ability of MHE to influence cardiac function in the zebrafish.

## Materials and Methods

Initially in this section parameters and techniques common to all experiments are discussed including zebrafish husbandry (1a) and the preparation of MHE (1b). We then describe the feeding regimen (2a) and data acquisition process (2b) for the automated hypercholesterolemia screen. Then a second set of experiments, performed with a different methodology than the hypercholesterolemia screen, are described for automated measurement of MHE's influence on cardiodynamics. For these experiments the feeding regimen (3a), data acquisition (3b), and computational algorithms (3c and 3d) employed in assessing cardiodynamic data are described. Finally a description of statistical tests utilized is provided (4).

### 1a. Zebrafish Husbandry

Adult zebrafish were housed in the jointUniversity of Cincinnati (UC)-Cincinnati Children's Hospital Medical Center (CCHMC) zebrafish facility. All zebrafish husbandry and experimental procedures were performed in accordance with and approved by theUC Institutional Animal Care and Use Committee (IACUC, protocol # 1D03020. Embryos were generated for this study from in-house lines of adult fish being bred, raised, and cared for according to established procedures [20]. Water conditions in this facility (pH = 7.1–7.4; temperature = 26.5–28.5°C; conductivity = 490–530 µS; and dissolved oxygen concentration = 5.0–7.5 mg L^−1^) were rigorously maintained through real-time computerized monitoring and dosing. For this study, transgenic *TG*(*kdrl:mCherry*) zebrafish with mCherry fluorescent protein driven by the cardiovascular specific kdrl promoter were crossed with a *casper* line containing a melanocyte/iridophore mutation [21]. The resulting double transgenic animals *TG(Kdrl:mCherry)/Casper* express red fluorescence in the vascular walls and are optically transparent through adulthood. In all data acquisition procedures fish were anesthetized in 125–150 mg L^−1^ MS-222 (tricaine) and mounted in 1.2% agarose in glass bottomed viewing slides.

### 1b. Preparing Hawthorn Extract

The leaves and flowers of *Crataegus laevigata*, obtained from Starwest Botanicals (Rancho Cordova, California), were crushed with mortar and pestle. Plant material was then weighed to 6.5 g and added to a 250 mL round bottom flask with Boileezer. Two-hundred mL ofmethanol was added to th flask and refluxed for 70 minutes. Filtrate was passed through Whatman 1 paper and solution was brought up to 250 mL with 80% methanol. This lead to a methanolic solution equivalent to 26 mg/mL pure plant product. Doses for administration in hypercholesterolemia screen were determined from an LD_50_ curve.

### 2a. Feeding for Automated Hypercholesterolemia Screen

For high-throughput analysis, 4 days post-fertilization (dpf) fish were fed a mixture that consisted of 2.5% v/v egg yolk in tank water in a method also described in [18]. After sonicating for 20 minutes at 5 minute intervals, 50 µM ezetimibe (Ryan Scientific) (from a stock concentration of 10 mg/mL in DMSO), or between 3.5–19.5 µg/mL methanolic extract of hawthorn leaves and flowers, combined with 2.5 µg/mL 23- (dipyrrometheneboron difluoride)-24-norcholesterol (BOD-CH. TopFluor, Avanti Polar Lipids) from 8 µL stock at a concentration of 0.3125 µg/µL in DMSO were added to the sonicated solution. The control solution was exactly the same except without ezetimibe or hawthorn extract. After incubation in food solution for 2 hours, fish were extracted from the treatments and allowed to swim in tank water overnight. The next morning fish were imaged as described below.

### 2b. Automated Hypercholesterolemia Screen

**Figure 1:**
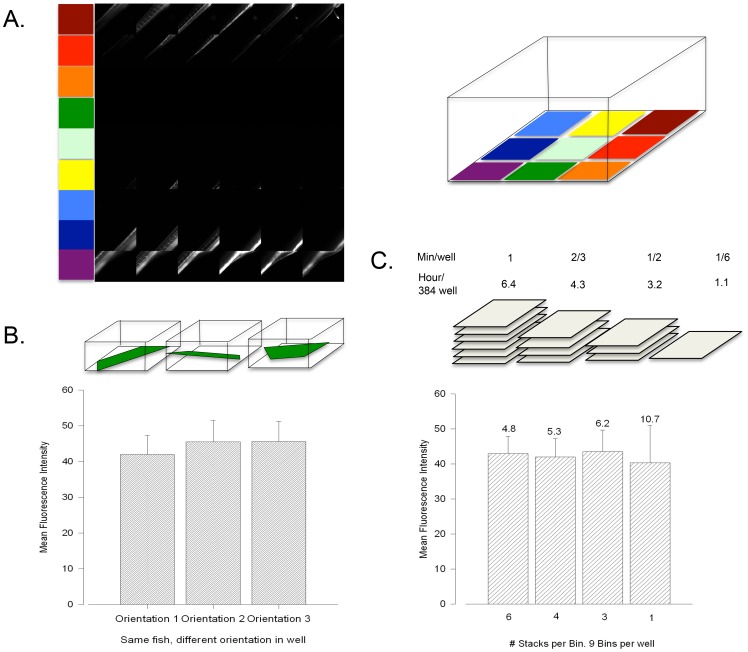
Hypercholesterolemia Screen Calibrations. Colors next to images correspond to those covering the area of a representation of a single well in a 38-well plate (upper right). B. In order to determine how a fish' orientation influences the measured fluorescence output, the same fish was measured in 3 different positions. C. Different numbers of slices per z-stack were taken of the same fish in the same position. This was to determine the number of stacks that lead to the least amount of error. Numbers above error bars are the values of the standard error of the mean. Above stack representations is the amount of time the Opera machine would takes to scan each well and an entire 384-well plate at the given number of z-slices per stack, assuming 9 stacks per well (as above).

**Figure 2:**
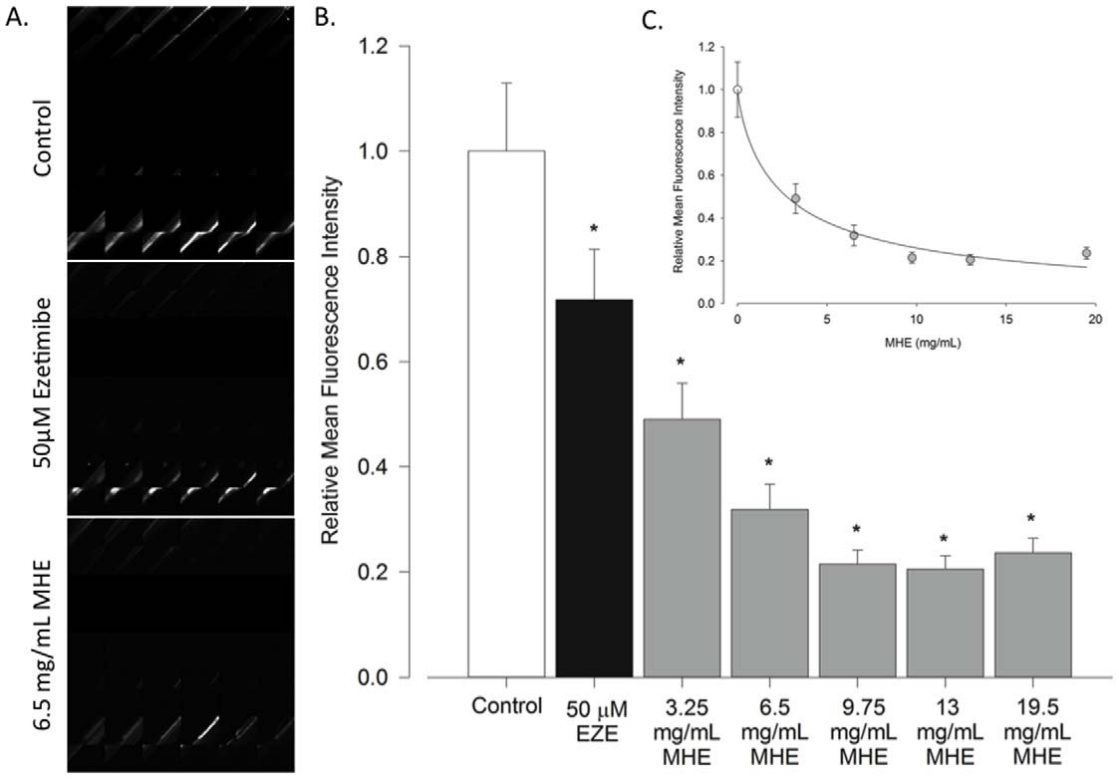
Automated Hypercholesterolemia Screen. A. Images of Control, 50 µM Ezetimibe treated, and 6.5 mg/mL methanolic hawthorn extract (MHE) treated 5 pf zebrafish embryos B. Quantified results of the automated screen. Bars represent the mean of the mean fluorescence intensity of each individual well (with values of 0, no reading, excluded). Control, 50 µMEzetimibe treated, and between 3.25 mg/mL and 19.5 mg/mL MHE treated groups are show. Ezetimibe served as a positive control C. Dose response curve illustrating the relationship between hawthorn dose and fluorescent output (R^2^ = 0.61). For this experiment n is between 13–30 per group.

Automated acquisition was performed in Perkin-Elmer's Opera high-throughput/high-content automated confocal system in 384-well plates with one anesthetized fish in each well in 20 µL of tank water. The Opera system scans a user-designated area of the well in the x-y direction and focuses on a user-defined displacement on the z-axis. Nine z-stacks were obtained at different x-y locations in each well and 6 z-slices were taken per stack in a total z-range of 250 µm at a spacing of 50 µm between each z-slice. This lead to 54 total images per well (Figure 1A). The orientation of the fish in each well was random. Mean fluorescence intensity from each group of 54 images (of one individual fish) was considered a data point, except in figure 1B and 1C, where error represents the error in the mean value of the group of 54 images. In all experiments utilizing the Opera system (figures 1 and 2) ImageJ was utilized for fluorescence quantification.

### 3a. Feeding for Heart Beat Measurement

Five dpf, Kdrl:casper fish were fed as above except that no ezetimibe or BOD-CH were administered and 6.5 mg/mL hawthorn extract was mixed into the egg solution. Fish were incubated in this mixture for two hours and removed to swim in tank water for 3 hours. After 3 hours, fish were anesthetized, mounted in agarose and imaged as described above.

### 3b. Heart Beat Acquisition

**Figure 3:**
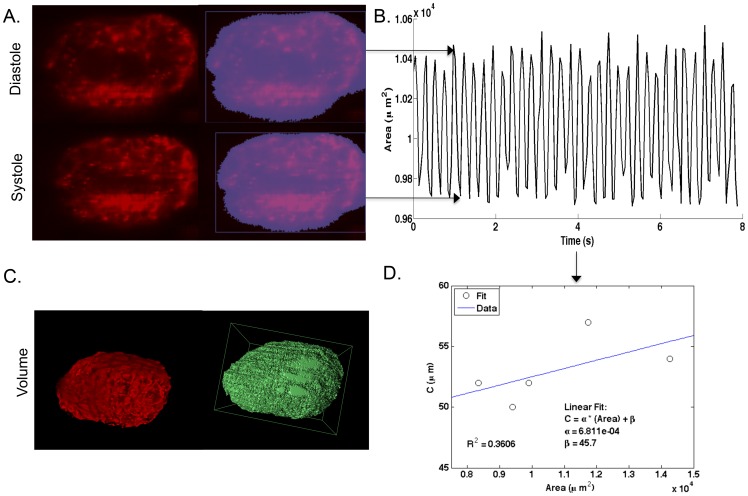
Heart Beat Detection and Area to Volume Conversion. A. Raw data and automated detection of area (A) of heart during diastole and systole. B. Cardiac waveform generated by automated detection of heartbeat (above) C. Measurement of the volume of chemically arrested hearts D. The C radius was calculated by correlating the volume of five arrested hearts to the cross-sectional areas of those hearts. This gave a relationship between the cross-sectional area and the C radius with the equation: C = (6.8×10^−4^) * A+46. Inputting this relationship into the equation for the volume of a prolate spheroid, V = (4/3)*π*x*y*z, where π*x*y = A and z = C, we get the relationship V = (4/3)A*C, where the volume of the ventricle is a function of the area measured. This equation is utilized to transform each area data point in B to volume measurements from which stroke volume (SV), heart rate (HR), cardiac output (CO) and ejection fraction (EF) are calculated (see figure 4).

Data was acquired of the heartbeat of 5 dpf zebrafish embryos embedded in agarose and oriented in a vertical position with the ventral side touching the bottom of the viewing slide. This orientation allows visual access to the ventricular chamber when viewing under an inverted microscope. Measurements were made with the Zeiss 7-Live Duo high-speed confocal system running the Zen software platform in the University of Cincinnati's Live Microscopy Core. The frame rate in these studies was 22.75 frames per second. Images were acquired for 8 . Automated detection of the acquired heartbeat over time was performed in Volocity. The program was instructed to detect a specific range of pixel intensity within each image. The range was empirically selected from a random subset of the data such that it corresponded well with the range occupied by the ventricle. The program was set to fill gaps in detected objects, and to exclude objects less than 1000 µm^2^ (Figure 3A).

### 3c. Relating Heart Area to Heart Volume

Five dpf Kdrl:casper fish were euthanized in 600 mg/L MS-222 in order to arrest the heart. Z-stacks were then acquired of the entire ventricular chamber in 5 fish. The average volume of the chambers was compared to the average area of a 2D image of the ventricle taken from a midpoint of each 3D stack and analyzed with Volocity image analysis software. The radius of the ventricle in the z axis (the C radius) was computed from these volume and area measurements assuming the shape of the ventricle to be a prolate spheroid. The correlation between the C radius of the ventricle and area of these five measurements yields the relationship C = (6.8×10^−4^) * A +46, where A is the area of the ventricle. This relationship allowed derivation of ventricular volume over time (see figure 3D for a detailed description).

### 3d. Automated Heart Beat Analysis

The results of the automated detection yielded time varying measurements of ventricular area over the heart beat cycle (Figure 3B). The ventricular area versus time data was then converted to ventricular volume versus time as described above in the section “Relating Heart Area to Heart Volume.” MATLAB was used to automatically extract the average change in volume of the left ventricle between systole and diastole using two different approaches. The first is solely based on the frequency-domain representation of the cardiodynamic data while the second uses both frequency- and time-domain data.

**Figure 4:**
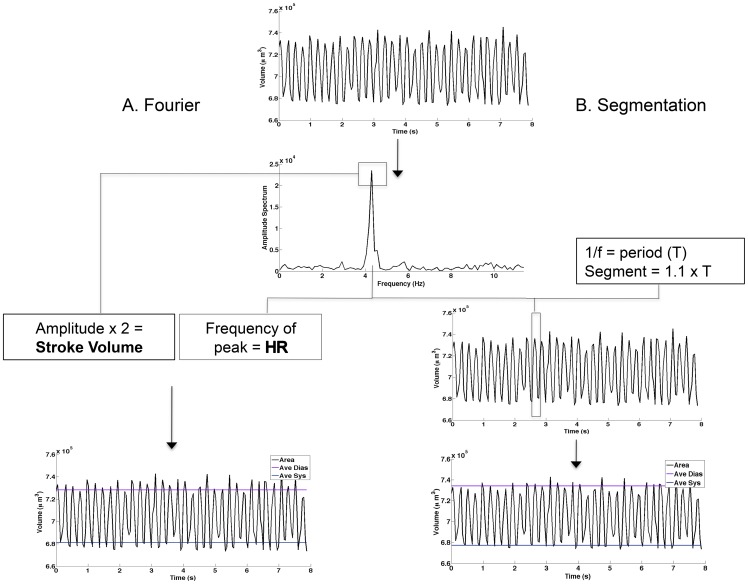
Waveform Analysis Methodologies. Volume change over time (top) calculated from area change as outlined in figure 3. Briefl, area waveform values were input into the equation, C = (6.8×10^−4^) * A + 46 from which volume over the heartbeat was calculated according to the equation V = (4/3)**A*C where A is the area of the ventricle during the beat cycle and C is the radius in the Z-direction. A. In the Fourier framework (left), a waveform is transformed to Fourier space in order to extract the amplitude and frequency (f) of the wave. In this case, these values represent ½ of the stroke volume (SV) and theheart rate (HR) respectively. From these parameters, we calculate cardiac output (CO) and ejection fraction (EF). A representative waveform with average diastolic and systolic volumes as calculated by Fourier is presented (bottom left). Notice that thedistance between diastole and systole compared to segmentation approach B. In th segmentation approach (right), the original waveform is transformed to Fourier space. The frequency of the peak of the transform is extracted to determine the period (T) of the waveform which is then utilized as a baseline value on which to base the size of segment for analysis. The algorithm measures maximum and minimum values within each segmen (which is sized at 1.1× T in order to increase the liklihood of capturing the maximum and minimum values) traversing the waveform. Stroke volume is calculated as the mean maximum value – mean minimum value and is represented as average diastole and average systole (bottom right).

The frequency-domain approach first mean subtracted each time-domain data set. The volume-time curve was then windowed using a Tukey tapered cosine window (r = 0.5) algorithm to minimize artifacts when subsequently converting to frequency-domain data using the fast Fourier transform (FFT). Proper normalization was performed on the frequency-domain to correct the amplitude for the curve length, sampling frequency, windowing, and measuring only the positive frequencies in the frequency-domain data [22]. The peak amplitude of magnitude of the normalized frequency-domain data was multiplied by two to obtain the peak-to-peak change in ventricular volume, which corresponds to the change between systole and diastole. The frequency corresponding the peak in the frequency-domain data was used as an estimate of the average heart rate during the eight-second acquisition (Figure 4A)

The frequency- and time-domain combined approach used the frequency domain data to estimate the heart rate as above (Figure 4A). The data was then portioned into segments equal to 110% of the heartbeat period, which assured that both systole and diastole occurred in each segment (Figure 4B). The global minimum and maximum ventricular volume was found for each segment. The average maximum and average minimum across segments was computed to obtain the average diastolic and systolic volume, respectively. The difference between these average volumes was computed and used to compute cardiac output and ejection fraction in a manner identical for both approaches. Figure S1 shows example volume-time curves and the average systolic and average diastolic volume using each of the above methods and a manual estimate

### 4. Statistics

Regression analyses and ANOVA tests were performed in SigmaStat software. P-values<0.05 were considered significant. Holm-Sidak post-hoc multiple comparison procedure was implemented for all ANOVA tests where significant differences were observed. Error bars represent the standard error of the mean.

## Results

### Automated hypercholesterolemia screen

In calibration experiments of the Opera automated high-content/high-throughput confocal system, we tested the variability in its measurement of fluorescent output. In order to determine the error in our studies introduced by variable orientation, we first tested how the automated system performed when the same fish was measured in 3 different orientations. Our results show that the mean fluorescent output is very similar when the same fish is measured in different orientations (figure 1B). Figure 1C shows that the standard error of the mean from the entire group of z-stacks taken in the well decreases with increasing slices per stack. The decrease was inversely proportional to the square root of the number of stacks, as would be expected from random error [23]. The estimated time for a scan of all 384 wells at different stack numbers is also shown in figure 1C.

The previous calibrations provided the background for our initial experiment with the Opera system, which was designed to test whether the setup could detect a difference between control and ezetimibe treatment, and also to test the ability of MHE to treat hypercholesterolemia in a dose-dependant manner. It was previously found that ezetimibe treatment at a concentration of 50 µM significantly decreased intravascular BOD-CH fluorescence [18], indicating that BOD-CH is absorbed in a manner similar to native CH and providing the positive control for our automated screen. Representative images of control, ezetimibe and MHE treated fish are shown in Figure 2A. The automated hypecholesterolemia screen was able to detect a difference between control and ezetimibe treated embryos (figure 2B). Also, Hawthorn treatment significantly reduced detected fluorescent output, even in the lowest-dose treatment group, and reduced fluorescent output in a dose-dependant manner, which suggests its efficacy in treating hypercholesterolemia (figure 2C).

### Automated Detection and Analysis of the Zebrafish Heart Beat

High-speed confocal microscopy combined with transgenic, transparent fish expressing tissue-specific fluorophores, provides an excellent tool with which to automate heart beat detection. The contrast between the heart and the surrounding tissue in the kdrl:casper transgenic line allows for relatively easy automated detection of the area encompassed by the cardiac endothelium over time. This detection method, represented in figure 3A, creates a cardiac waveform, figure 3B, which can subsequently be analyzed for aspects pertaining to cardiac performance (see figure 4 for explanation of analysis algorithm). In order to calculate stroke volume (SV) from this time-varying area data, it is necessary to test the relationship between the area of the heart and its actual volume. This relationship was determined in five fish by stopping the heart, measuring the area, then measuring the total volume of the heart (figure 3C). From these data, we derived a linear relationship between the radius in the z-plane (denoted as the variable C) of our images and the area as measured in our detection procedure (figure 3D). We utilized this relationship to convert changes in ventricular cross-sectional area to estimates of ventricular volume over the beat cycle.

**Figure 5:**
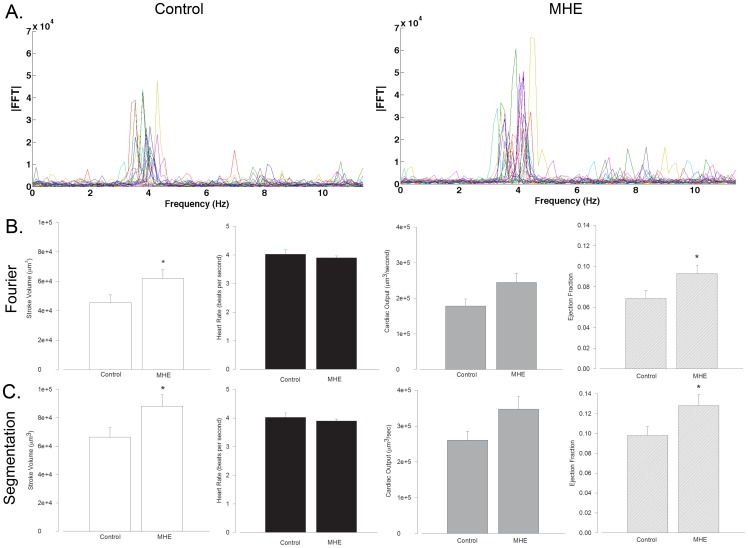
Cardiodynamic Influence of Methanolic Hawthorn Extract (MHE). A. Fourier transformed data of control and 6.5 mg/mL MHE treated 5dpf zebrafish embryos. Notice the increased amplitude in the MHE treated fish which corresponds to an increased stroke volume (SV) B. and C. SV, heart rate (HR), cardiac output (CO) and ejection fraction (EF) measurements for Fourier method (B) and segmentation method (C). SV and EF were significantly increased according to both measurement paradigms (* indicates P<0.05, n = 22, 26 for Control and MHE treated fish respectively).

Combined with our initial hypercholesterolemia screen, this automated detection procedure further streamlines the drug-discovery and toxicity testing process. In order to demonstrate the utility of this methodology, we tested the influence of a dose of MHE that was effective in our hypercholesterolemia treatment screen (6.5 mg/mL) on cardiodynamics and analyzed the data using both of the automated methods. According to both analysis paradigms, the results indicate an increase in SV and EF in hawthorn treated fish compared to untreated controls, indicating enhanced cardiac function after hawthorn treatment (figure 5).

## Discussion

The purpose of this study was to create a platform in which to rapidly test functional food-based treatments of disease. This platform can also test single-molecule treatments of disease as evidenced by the results of ezetimibe treatment (figure 3B). The initial hypercholesterolemia screen concentrated on a simple output metric: mean fluorescence intensity as judged from the entirety of images collected in each well. The simplicity of this measurement procedure allows our methodology to be applicable to many other confocal systems, and many other image analysis programs. This simplicity also decreases the computational demand of image analysis. The large data sets generated in high-throughput/high-content screening place a large burden on most computer systems. Much more demanding however, is complicated data analysis. Our system allows researchers to initially screen and detect changes with simple analysis, building on this analysis if necessary.

In our initial hypercholesterolemia screen we were able to detect a decrease in mean fluorescence intensity in ezetimibe treated fish compared to controls. However, this difference is not as substantial as the difference between control and ezetimibe treatment in our manual screen [18]. The reason for this discrepancy is likely that in the automated Opera screen, data from the entire fish is acquired, including from the gut. As fish appear to eat a similar amount of food between treated groups and controls, this creates similar fluorescence output in the digestive tract in these groups. Therefore, it is likely that our assessment of mean fluorescence intensity over the entirety of collected images in each well le to this discrepancy between the magnitude of ezetimibe's effect between manual and automated screen. The effect of fluorophore in the gut was minimized by allowing a 16 hour interval between feeding and imaging where fish had no access to food. This was to ensure that a maximal amount of fluorophore was expelled from the gut before imaging. Also, the area of the well imaged was optimized to capture as little of the intestine as possible. The influence of fluorescent cholesterol in the gut nevertheless has potential to decrease the sensitivity of the screen. However, this occurrence is also beneficialin its potential to identify both compounds that decrease intravascular cholesterol levels by inhibiting cholesterol absorption and compounds that facilitate the expulsion of cholesterol. Hawthorn extract had a dramatic effect on BOD-CH fluorescent output compared to controls and in a dose-dependant fashion (figure 2B and 2C). This agrees with our longer-term studies to determine the effect of whole ground hawthorn leaves and flowers on intravascular cholesterol levels [18].

Previous attempts to automate the analysis of cardiodynamic data in zebrafish employed the analysis of brightfield images of the heart beat [24] and have derived measurements of heart beat rhythmicity from Fourier power spectrum representations of blood flow in the caudal vasculature [25]. Compared to brighfield imaging, high-speed confocal data has the advantage of providing high contrast between the organ and surrounding tissue, greatly simplifying the automated detection of heart movements. Analyzing the heart beat for cardiodynamic data with the method presented here, opposed to extracting cardiac parameters from measurements in the vasculature [25], allows the extraction of not only frequency measurements, but also measurements of stroke volume and ejection fraction which indicate the inotropic state of the heart. However, the rhythmicity analysis presented in [25] provides a powerful tool for detecting the arrhythmic effects of drugs.

In order to validate our two automated analysis methods, we tested the accuracy of both techniques in determining stroke volume compared to manually measured waveforms (quantified by measuring the peak and trough of each wave in a dataset and subtracting the mean maximum from the mean minimum). Two different datasets were analyzed in these measurements—one from a healthy heart and one from an erratically beating heart (Figure S1). The results demonstrate that in both cases, the segmentation approach based on frequency- and time-domain analysis better predicts manual measurements. While the methods yield different absolute values of SV, their ability to detect changes in these parameters is nearly identical (figure 5 and 5c). These methods of analysis therefore verify one another in their ability to detect the effects of cardiotonic agents. Also, the accuracy of both increases as more data is obtained, however the Fourier domain approach requires the data to be recorded over many cardiac cycles while the segmentation approach can be computed from as little as 1 cardiac cycle. The Fourier domain approach also effectively linearizes the cardiac waveform data, resulting in a smaller measure of the average change in volume over the cardiac cycle. Conversely, the segmentation approach is more susceptible to noise, though by averaging over many heart beats this effect is minimized.

Littleton *et al*, 2012 showed that cardiac output decreases with increasing cholesterol, and that there is a significant difference in CO between 0.1% CH in the diet compared to 8% CH in the diet. This data was utilized to compare the manual measurement of CO from Littleton *et al*, 2012 to our automated methods (Figure S2). CO was analyzed with both the Fourier and segmentation approaches and compared to manual analysis. As in manual measurements, both automated methods detected a significant difference between the lowest treatment group (0.1% CH) and the highest treatments group (8% CH). When compared to the manual methodology, the segmentation approach lead to a similar correlation between CH and CO as indicated by analogous R^2^ values, as well as similar slopes (Figure S2. This demonstrates congruous strength of detected effect of CH on CO and similar sensitivity in the analysis paradigms. The Fourier approach shows slightly decreased R^2^ and slope value. The error in the measurements in the Fourier analysis is however less than both manual and segmentation approaches.

Longer-term dietary intervention in zebrafish in a previous study [18] indicated that when ground hawthorn leaves and flowers were added to food combined with cholesterol there was an interaction effect between hawthorn and cholesterol to improve cardiac output compared to cholesterol treated fish. In this study, treatment with MHE lead to a statistically significant increase in SV and EF. These results therefore agree with one another, as our method in this present endeavor required that we introduce hawthorn in an egg yolk and water solution. Also, the approximately 20% increase in SV and EF due to MHE treatment suggests it to be a clinically relevant inotropic agent, supporting its present use as a cardiotonic in heart failure [26]. Previous studies on alcoholic extracts of hawthorn leaves and flowers demonstrate increased cardiomyocyte contractility and vasodilatory effects in cell culture and *ex vivo* experimental paradigms [26]. It is therefore likely that the increase we detected in SV and EF is due to a combination of vasodilation and increased cardiac inotropy, combining to improve cardiac function.

In this study we have provided a simple and robust platform for testing the efficacy and side effects of dietary intervention of hypercholesterolemia in an *in vivo*, highly-automated screen. We have also confirmed that methanolic extract of *Crataegus laevigata* is likely an antihypercholesterolemic treatment, as well as a potential cardiotonic agent. This indicates that this plant has wide ranging, holistic influence on bodily functionand that more research needs to be done in order that its proper indication in disease is elucidated.

## Supporting Information

Figure S1 **Comparison of Segmentation and Fourier Analysis Methods.** Healthy (upper) and erratic (lower) waveforms were analyzed in order to determine which method best detected peaks and troughs in each case. In both cases the segmentation approach gave closer values to manual measurement than did the Fourier transform approach. Lines represent mean systole (blue) and mean diastole (purple) as calculated with each method.
(TIF)

Figure S2 **Manual and Automated Analyses of Cholesterol (CH) vs. Cardiac Output (CO) Regression.** Comparison of regression characteristics between manual, segmentation and Fourier approaches. R^2^ represents the strength of correlation between the variables. Slope demonstrates the detected magnitude of impact of CH on CO. *indicates P<0.05 between 0.1% CH (lowest dose) and 8% CH (highest dose). This difference was detected in each trial. Data for analyses utilized with permission from Littleton *et al*, 2012 [18].
(TIF)
