# Supplementary figures and images for: Automated In Vivo Platform for the Discovery of Functional Food Treatments of Hypercholesterolemia
Source: PLoS One. 2013 Jan 21;8(1):e52409. doi: 10.1371/journal.pone.0052409 (PMC3549914; doi:10.1371/journal.pone.0052409)

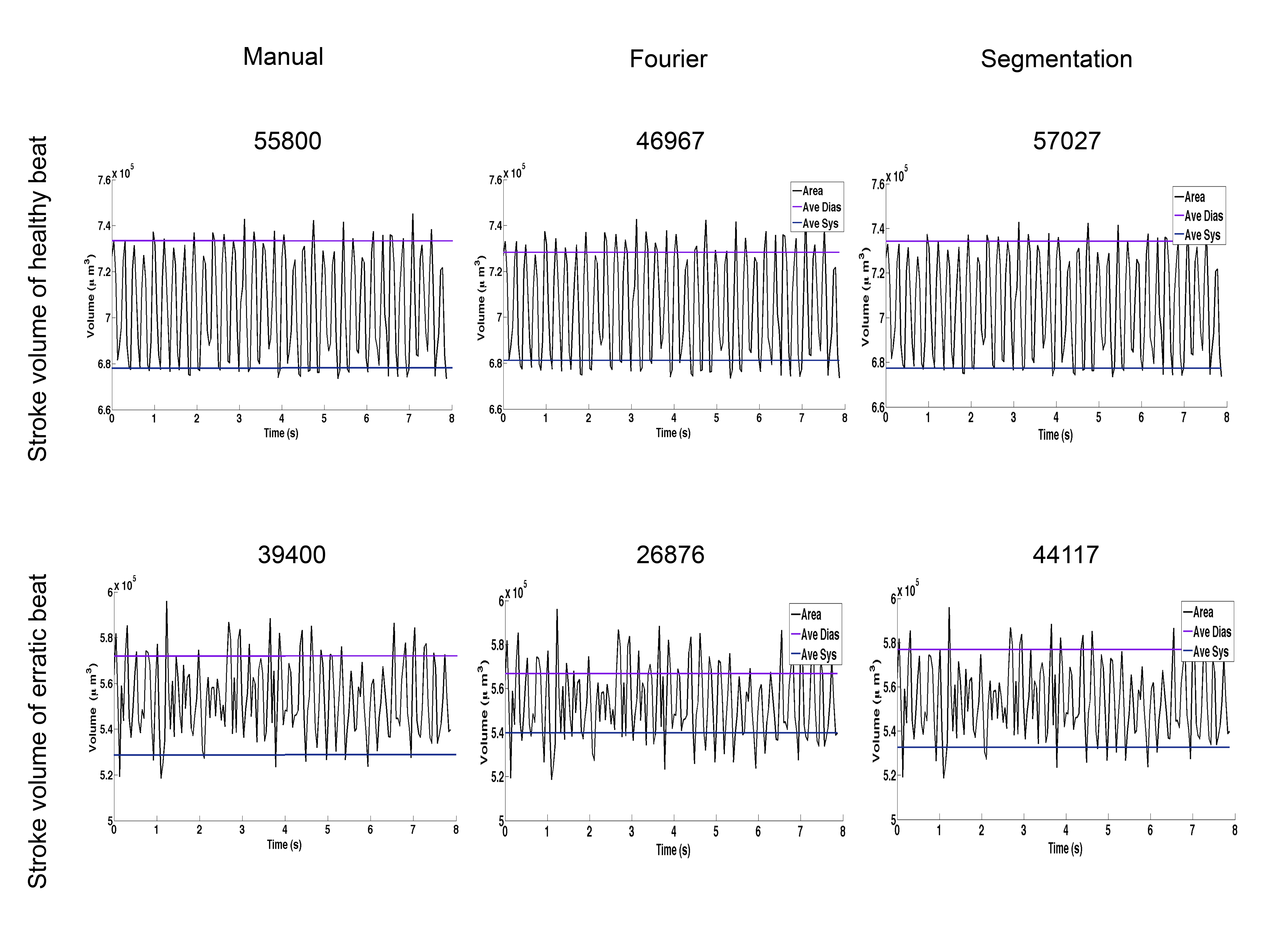

Supplement: Figure S1 — Comparison of Segmentation and Fourier Analysis Methods. Healthy (upper) and erratic (lower) waveforms were analyzed in order to determine which method best detected peaks and troughs in each case. In both cases the segmentation approach gave closer values to manual measurement than did the Fourier transform approach. Lines represent mean systole (blue) and mean diastole (purple) as calculated with each method. (TIF) [file pone.0052409.s001.tif]
